# Enhanced Sound Absorption of Aluminum Foam Composites by Introducing Pore-Penetrating Fibers

**DOI:** 10.3390/ma18245515

**Published:** 2025-12-08

**Authors:** Bei Huang, Shuang Xiong, Xin Wang, Longyue Qin, Xiaoqing Zuo, Hui Wang

**Affiliations:** 1 Faculty of Materials Science and Engineering, Kunming University of Science and Technology, Kunming 650093, China; hbeing003@163.com (B.H.); sdlgdxwangxin@163.com (X.W.); 18135194475@163.com (L.Q.); 2 State Key Laboratory for Advanced Metals and Materials, University of Science and Technology Beijing, Beijing 100083, China; xs17614025754@163.com; 3 School of Green and Advanced Technologies, Nanning Vocational and Technical University, Nanning 530008, China

**Keywords:** pore-penetrating 316L stainless steel fibers composited aluminum foam (PPFCAF), pore structure, sound absorption valley, sound absorption performance, sound absorption mechanism

## Abstract

To address the issue of sound absorption valleys in open-cell aluminum foam and enhance mid-to-high frequency (800–6300 Hz) performance, we developed a novel pore-penetrating 316L stainless steel fiber–aluminum foam (PPFCAF) composite using an infiltration method. The formation mechanism of the pore-penetrating fibers, the resultant pore-structure, and the accompanying sound absorption properties were investigated systematically. The PPFCAF was fabricated using 316L stainless steel fiber–NaCl composites created by an evaporation crystallization process, which ensured the full embedding of fibers within the pore-forming agent, resulting in a three-dimensional fiber-pore interpenetrating network after infiltration and desalination. Experimental results demonstrate that the PPFCAF with a porosity of 82.8% and a main pore size of 0.5 mm achieves a sound absorption valley value of 0.861. An average sound absorption coefficient is 0.880 in the target frequency range, representing significant improvements of 9.8% and 9.9%, respectively, higher than that of the conventional infiltration aluminum foam (CIAF). Acoustic impedance reveal that the incorporated fibers improve the impedance matching between the composite material and air, thereby reducing sound reflection. Finite element simulations further elucidate the underlying mechanisms: the pore-penetrating fibers influence the paths followed by air particles and the internal surface area, thereby increasing the interaction between sound waves and the solid framework. A reduction in the main pore size intensifies the interaction between sound waves and pore walls, resulting in a lower overall reflection coefficient and a decreased reflected sound pressure amplitude (0.502 Pa). In terms of energy dissipation, the combined effects of the fibers and refinement increase the specific surface area, thereby strengthening viscous effects (instantaneous sound velocity up to 46.1 m/s) and thermal effects (temperature field increases to 0.735 K). This synergy leads to a notable rise in the total plane wave power dissipation density, reaching 0.0609 W/m^3^. Our work provides an effective strategy for designing high-performance composite metal foams for noise control applications.

## 1. Introduction

Open-cell aluminum foam features a vast network of interconnected pores, with its interconnected skeletal framework offering unique physical and functional properties [1,2]. It is characterized by low density, high specific strength, excellent electrical and thermal conductivity, and a high specific surface area [3]. Open-cell aluminum foam is widely regarded as an ideal material for noise absorbers, electromagnetic absorbers, heat dissipators, filters, and catalysts [4]. The sound absorption properties of open-cell aluminum foam have been extensively studied [5,6,7,8]. Compared to porous materials such as glass wool, rock wool, and polyurethane foam, open-cell aluminum foam exhibits superior fire resistance, water resistance, and environmental sustainability. It can effectively mitigate mid-to-high frequency noise environments in transportation, construction, and aerospace sectors.

The sound absorption curve fluctuates as frequency increases. This common phenomenon is caused by standing wave effects [9]. When sound waves interact with a material, a portion of the wave penetrates to the bottom and is reflected by the rigid backing. This reflected wave superimposes with the incident wave, continuing to propagate from the surface and forming a standing wave within the material layer. The resonance frequency at one-quarter wavelength corresponds to the absorption peak, while the antiresonance frequency at one-half wavelength corresponds to the absorption valley. These resonance and antiresonance frequencies are jointly determined by the material thickness and the complex wave number of the sound wave within the material (closely related to microscopic parameters such as flow resistance, porosity, and tortuosity). However, further research indicates that porous materials with different pore structures exhibit varying sound absorption fluctuation curves [10]. Compared to network-like, fibrous, and granular porous materials, cellular aluminum foam exhibits more pronounced fluctuations in its sound absorption curve. The microstructures of different materials cause varying damping attenuation. Porous materials with high flow resistance and tortuosity in their pore structures create more friction opportunities for sound waves against pore walls, leading to increased viscous losses. This results in broader resonance peaks and filled absorption valleys. In contrast, cellular aluminum foam shows sharper sound absorption peaks and valleys due to its relatively low flow resistivity.

One of the main strategies for further expanding the formant and filling the sound-absorbing valley involves modifying the pore morphology and complexity of the foam material. Huang et al. [11] found that aluminum foam with a double main pore-porous cell wall structure increases the pore wall surface area, effectively enhancing pore complexity and reducing interface reflections between sound waves in the absorption valley region and the rigid backing. Otaru et al. [12] used finite element simulations to show that altering pore structure parameters reduced the sound absorption valley in CIAF. However, they did not conduct a detailed analysis of the underlying improvement mechanism. Nonetheless, this approach offers only limited improvement because of the highly interconnected open-cell structure. As a result, adding more media, such as fibers, polyurethane, or aerogels, to the pore structure can also diminish acoustic wave reflections. Ren et al. [13,14] demonstrated that synthesizing ZnO or CO_3_O_4_ needle-like crystals on the pore walls of open-cell aluminum foam increased the sound absorption coefficient by up to approximately 40%. Dong et al. [15] successfully created a new aluminum-polyurethane interpenetrating phase composite foam, showing improved sound absorption performance. However, current research on the sound absorption properties of foam structures mainly focuses on overall absorption curves and peaks, with few reports on sound absorption valleys.

Considering the stability of composite structures, previous studies [16,17] created stainless steel/fiber-reinforced aluminum foam by combining fibers with pore-forming agents. These studies found that the fiber-reinforced aluminum foam exhibited a 7.2% increase in sound absorption valley values. This suggests that the addition of fiber can enhance sound absorption. However, a few fibers are introduced into the pore spaces, their penetration into the foam is limited, resulting in a modest impact on sound absorption.

This paper presents a novel approach to producing fiber–NaCl composites through evaporation crystallization, successfully embedding 316L stainless steel fibers entirely within the pores by infiltration casting to create a composite structure with fibers penetrating the foam pores. The study investigates the effects of pore structure parameters—including pore-penetrating fiber diameter (15–30 μm) and main pore size (0.5–0.8 mm)—on the pore structure and sound absorption performance (sound absorption valleys) of PPFCAFs. Subsequently, finite element simulations were employed to analyze the acoustic behavior of the PPFCAFs. By applying finite element analysis methods, numerical studies were performed on acoustic parameters such as reflected sound pressure and total sound energy dissipation, further clarifying the synergistic mechanism of pore-penetrating fibers in affecting sound reflection and sound absorption.

## 2. Preparation and Methods

### 2.1. Preparation

The primary materials used to prepare the PPFCAFs through infiltration casting were the pore-forming agent and the matrix alloy. This study employed a 316L stainless steel fiber–NaCl composite as the pore-forming agent and AlSi12 as the matrix material.

#### 2.1.1. Design of 316L Stainless Steel Fiber–NaCl Composite

The precursor is the pore template for preparing infiltration aluminum foam and is the key to controlling the pore structure morphology. Previous studies have found [16] that only a few fibers were introduced into the pores in a precursor prepared by mechanically mixing fibers with preform particles. Therefore, improving the fiber-penetrating porosity has become a technical difficulty in the preparation.

**Figure 1:**
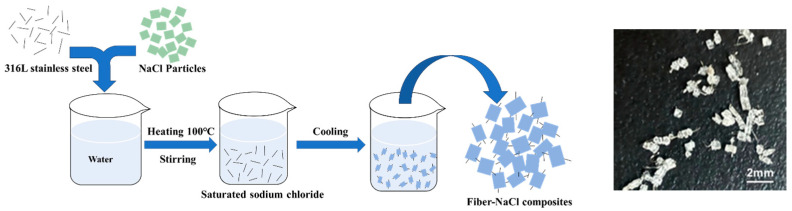
Preparation and picture of stainless steel 316L stainless steel fiber–NaCl composites.

In this work, an evaporation crystallization was ingeniously employed to prepare a fiber–NaCl particle composite. NaCl (DSCG, Dalian, Liaoning, China) was selected as the raw material for the pore-forming agent. Two fiber diameters (15 μm and 30 μm) of 316L stainless steel fibers (Yutong, Shanghai, China) with a length of 3 mm were chosen for the composite. The fiber–NaCl composites were prepared by the evaporation crystallization method. The specific preparation process is shown in Figure 1. First, NaCl particles were dissolved in water to obtain a supersaturated solution of NaCl. Subsequently, 316L stainless steel fibers were immersed in the saturated NaCl solution. NaCl was nucleated and crystallized on the fiber’s surface, and the NaCl coating grew around the fibers as water vapor evaporated, forming the fiber–NaCl composites. Unlike the preforms used in previous studies, the fibers were embedded entirely within the NaCl particles, creating conditions for the subsequent formation of a fiber-permeated pore structure.

#### 2.1.2. Preparation of PPFCAF

**Figure 2:**
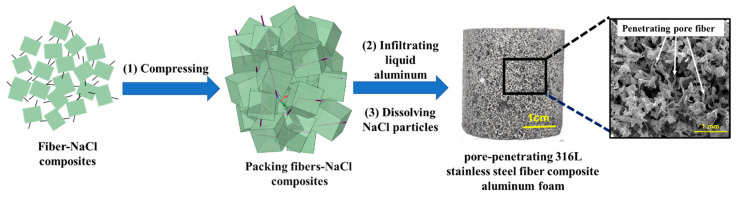
Schematic diagram of the preparation for PPFCAF.

PPFCAF was prepared through infiltration casting, which involved the cold pressing of fiber–NaCl composites, heating, infiltration of an AlSi12 melt, and desalination of the NaCl particles. Figure 2 is a schematic diagram of the specific preparation process. This study prepared PPFCAFs with different fiber diameters (15 μm and 30 μm) and main pore sizes (0.5–0.8 mm). Table 1 shows the specific parameters of the samples. Conventional infiltration aluminum foam (CIAF) with a main pore size of 0.5 mm was also prepared for comparison. PPFCAFs and CIAF were processed into cylindrical samples with a size of 30 mm and a thickness of 30 mm.

### 2.2. Test Methods

#### 2.2.1. Determination of Pore-Penetrating Fibers

Since the pore-penetrating fibers in the pore structure directly affect sound absorption performance, it is essential to quantify the fiber content within the fiber–NaCl composites. Fiber–NaCl composites with different NaCl particle sizes (0.8 mm, 0.7 mm, 0.6 mm, and 0.5 mm) were weighed in 5 g separately. These composites were placed into beakers, and the NaCl particles were dissolved. This dissolution and filtration procedure was repeated twice to ensure complete removal of the salt. The remaining fiber residue was then transferred to a drying oven. After drying, the fibers were weighed to determine their mass. The fiber volume fraction was calculated using Equation (1), and the fiber count was determined using Equation (2). Table 2 presents the fiber content and mass of fiber–NaCl composites. As shown in Table 2, the fiber content in the composites ranged from 0.67% to 0.85% vol. As the size of the NaCl particles decreased, the volume fraction of fibers within the fiber–NaCl composites decreased. This happens because the length of the large pore-penetrating fibers matches that of the cubic salt particles. The number of pore-penetrating fibers increases for fiber–NaCl composite particles with smaller main pore sizes, despite having a lower fiber volume content *V_f_*.

(1) $$V_{f}=\frac{M_{f}\rho_{f}}{\left(M_{t}-M_{f}\right)\rho_{NaCl}}\times 100\%$$

where *M_f_* represents the mass of the pore-penetrating fibers, *ρ_f_* denotes the density of the 316L stainless steel fiber (7.80 g/cm^3^), *M_t_* indicates the mass of the fiber–NaCl particle composite, and *ρ_NaCl_* signifies the density of the NaCl particles (2.167 g/cm^3^).

(2) $$N=\frac{M_{f}}{L_{t}\pi r^{2}\rho_{f}}$$

In the formula, *L_f_* represents the length of the fiber, with a value of 3 mm, and *r* denotes the fiber diameter.

#### 2.2.2. Characterization of Microscopic Pore Structure

The microstructure of the samples, including the distribution of pore-penetrating fibers and the pore structure morphology of the PPFCAFs, was observed using a scanning electron microscope (VEGA3, TESCAN, Brno, Czech Republic) and a digital magnifier. ImageJ software 1.53kwas used to statistically analyze the SEM images, determining the average main pore size *D* and the average cell wall pore size *d* for the PPFCAF with single-pore sizes.

The porosity of open-cell aluminum foam *ϕ* was determined using the direct density measurement method [18,19] and calculated using Equation (3).

(3) $$\phi =\left(1-\frac{\rho_{foam}}{\rho_{AlSi2}}\right)\times 100\%$$

where *ρ_foam_* denotes the apparent density of the PPFCAFs, while *ρ_AlSi12_* represents the solid density of the AlSi12 aluminum alloy; the AlSi12 aluminum alloy density was determined using the displacement method.

#### 2.2.3. Acoustic Performance

According to the Chinese standard GB/T 18696.2-2002 [20], the sound absorption coefficient and acoustic impedance were measured in the frequency range of 800–6300 Hz using an acoustic impedance tube (SW477, BSWA TECH, Beijing, China). For this purpose, the test samples were cylindrical, with a thickness of 30 mm and a diameter of 30 mm, as specified in Table 1. The impedance tube simulated a rigid acoustic backing condition, and the data analysis was based on the transfer function method. The average value of the absorption curve of the sample in the frequency range of 800–6300 Hz was taken as the average absorption coefficient of the sample. The maximum value of the absorption curve in the frequency range of 800–2500 Hz was determined as the sound absorption peak, and the minimum value in the frequency range of 2500–5000 Hz was defined as the sound absorption valley. The specific test process is described in the Supplementary Materials.

### 2.3. Finite Element Method (FEM)

This study employed Pressure Acoustics and Thermoviscous Acoustics modules within COMSOL Multiphysics software 5.3a to model the acoustic properties of the samples. First, simulations were conducted on the equivalent fluid model of PPFCAFs to analyze its acoustic behavior, including the reflected sound pressure field and total sound energy dissipation distribution across the sound absorption valley frequency range. Finally, an ideal geometric model was utilized to observe sound wave propagation.

#### 2.3.1. Establishment of the Equivalent Fluid Model

Porous metallic materials, such as aluminum foam, create rigid frameworks that allow their sound absorption characteristics to be modeled using equivalent fluid approaches. The geometric model was divided into a fluid (porous medium) domain, an air (sound pressure) domain, and a perfectly matched layer. The simulation was conducted within the Pressure Acoustics (frequency domain) module. In this work, the Wilson acoustic model [21] was employed to model the acoustic characteristics of PPFCAFs. The expression of Wilson’s acoustic model is defined by Equations (4)–(9).

(4) $$\rho_{\mathrm{eq}}=\rho_{\infty }\frac{{\left(1+j\omega \tau_{vor}\right)}^{1/2}}{{\left(1+j\omega \tau_{vor}\right)}^{1/2}-1}$$

(5) $$K_{eq}=K_{\infty }\frac{{\left(1+j\omega \tau_{\mathrm{ent}}\right)}^{1/2}}{{\left(1+j\omega \tau_{\mathrm{ent}}\right)}^{1/2}+\gamma -1}$$

(6) $$\rho_{\infty }=\frac{\rho_{0}\alpha_{\infty }}{\phi }$$

(7) $$K_{\infty }=\frac{\gamma p_{0}}{\phi }$$

(8) $$\tau_{\mathrm{vor}}=\frac{2\rho_{0}\alpha_{\infty }}{\phi \sigma_{f}}$$

(9) $$\tau_{\mathrm{ent}}=\mathrm{Pr}\tau_{\mathrm{vor}}$$

where *ρ*_eq_ is the equivalent density. *ρ_∞_* is high frequency dynamic density, *j* is the imaginary unit, *ω* is angular frequency, *K_∞_* is body modulus of high frequency limit, and *τ_ent_* is entropy model relaxation time. *K_eq_* is the equivalent bulk modulus. *τ_vor_* is the vortex mode relaxation time, and *γ* is the specific heat ratio. *ρ_∞_* is the high-frequency limit of dynamic density, α_∞_ is the tortuosity, and *p_0_* is the pressure of the basic state. σ*_f_* is flow resistivity, *ϕ* is the porosity of foam, and *Pr* is the Prandtl constant.

Required parameters were obtained through experimental testing (porosity *ϕ*, flow resistivity σ*_f_*) or theoretical calculation (tortuosity *α_∞_*). This Wilson model introduces two key input parameters: flow resistivity and tortuosity. Specific details regarding their determination were provided in the Supplementary Materials.

**Figure 3:**
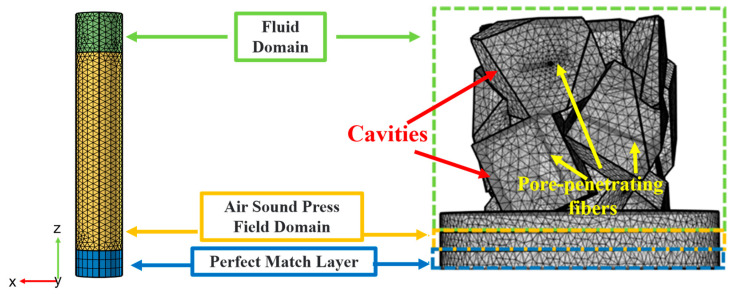
Geometric mesh partitioning for the equivalent fluid model (**left**) and the ideal fluid model (**right**).

The simulation process is conducted in the Pressure Acoustics (Frequency Domain) module. The fluid domain (porous medium domain) was set as a porous medium acoustic interface, with the Wilson acoustic model selected for the porous medium acoustic model. The fluid properties were set as air, while the porous matrix properties were defined as material properties, with the porous matrix approximated as rigid. The air domain was defined as the background sound pressure field, with the pressure type set as a plane wave propagating along the *z*-axis toward the surface of the porous medium. The sound pressure was 1 Pa, and the temperature was 293.15 K. All interfaces were configured as rigid sound field boundaries, with an initial pressure value of 0. Both the fluid domain and air domain employed free tetrahedral meshing, while the perfect matching layer utilizes swept hexahedral meshing. The maximum grid unit size was one-sixth of the maximum acoustic frequency, the minimum unit size was 2.1 × 10^−5^ mm, the maximum element growth factor was 1.3, the tortuosity factor was 0.3, and the narrow region resolution was 0.5. The structural mesh partition is shown in Figure 3 (left).

#### 2.3.2. Establishment of the Ideal Geometric Model

An ideal geometric model was employed to simulate sound wave propagation within the pores of PPFCAFs. The model encompassed the distribution of sound velocity and temperature within the pores, as well as the influence of pore-penetrating fibers on sound transmission.

Unlike earlier studies [16] that used ideal models with sparse fiber embeddings, this PPFCAF design incorporated pore-penetrating fibers throughout its pore network. The resulting ideal porous structure was made of stacked polyhedra embedded with these fibers. These polyhedra touched each other, creating interconnected three-dimensional cellular networks. The geometric model comprised three components: the fluid domain (representing the porous medium), the air sound pressure field, and the perfectly matched layer. Figure 3 (right) illustrates the PPFCAF geometric model with a pore size of 0.5 mm.

Because the ideal geometry simulated the acoustic behavior inside a cavity, the entire geometric domain was modeled as an air medium. The simulation used the Thermo-Viscoelastic Acoustics (Frequency Domain) module. The thermoviscoelastic acoustic model is grounded in the conservation laws of fluid mechanics [22], including mass, momentum, and energy conservation. Equations (10)–(15) detail these specific laws.

(10) $$i\omega \rho_{t}+\nabla \cdot \left(\rho_{0}u_{t}\right)=0$$

(11) $$i\omega \rho_{0}u_{t}=\nabla \cdot \left\{-\rho_{t}I+\mu \left(\nabla u_{t}+{\left(\nabla u_{t}\right)}^{T}\right)-\left(\frac{2}{3}\mu -\mu_{B}\right)\left(\nabla \cdot u_{t}\right)I\right\}$$

(12) $$\rho_{0}c_{p}\left(i\omega T_{t}+u_{t}\cdot \nabla T_{0}\right)-\alpha_{p}T_{0}\left(i\omega p_{t}+u_{t}\cdot \nabla p_{0}\right)=\nabla \cdot \left(k\nabla T_{t}\right)+Q$$

(13) $$\rho_{t}=\rho_{0}\left(\beta_{T}p_{t}-\alpha_{p}T_{t}\right)$$

(14) $$\alpha_{p}=\frac{1}{c_{0}}\sqrt{\frac{c_{p}(\gamma -1)}{T_{0}}}$$

(15) $$\beta_{T}=\frac{1}{\rho_{0}}\frac{\gamma }{c_{0}^{2}}$$

where *ϖ* represents angular frequency, *j* is the imaginary unit. *ρ_t_* represents the air density perturbation, *ρ*_0_ represents the air density at standard temperature and pressure, ***u_t_*** represents the air velocity vector, ∇ and represents the Laplace operator. ***T*** represents the transpose symbol. *μ* is the dynamic viscosity of air, ***I*** represents the identity matrix, *μ_B_* is the bulk viscosity of air, and *c_p_* is the specific heat at constant pressure. *T_t_* is the temperature of the air medium in the porous medium, and *T*_0_ is the air temperature under standard conditions. *α_p_* is the thermal expansion coefficient, *p_t_* is the disturbance of pressure, and *p*_0_ is the pressure of the basic state. *k* is thermal conductivity. *Q* is the volumetric heat source term. *β_T_* is the compressibility factor, and *c*_0_ is the sound speed. *γ* is the specific heat ratio.

The fluid properties were set to air. Sound waves propagated along the *Z*-axis toward the porous medium surface with a sound pressure of 1 Pa and a temperature of 293.15 K. The background sound pressure field fluid was set to air, with corresponding parameters configured according to air properties at 293.15 K. All interfaces were defined as rigid acoustic boundaries. The entire geometry was meshed using a free tetrahedral mesh, with a minimum element mass of *c/6f* and an average element mass of 0.6. The acoustic simulation frequency spanned 800 to 6300 Hz.

## 3. Results and Discussion

### 3.1. Pore Structure

#### 3.1.1. Influence of Pore-Penetrating Fibers on Pore Structure

**Figure 4:**
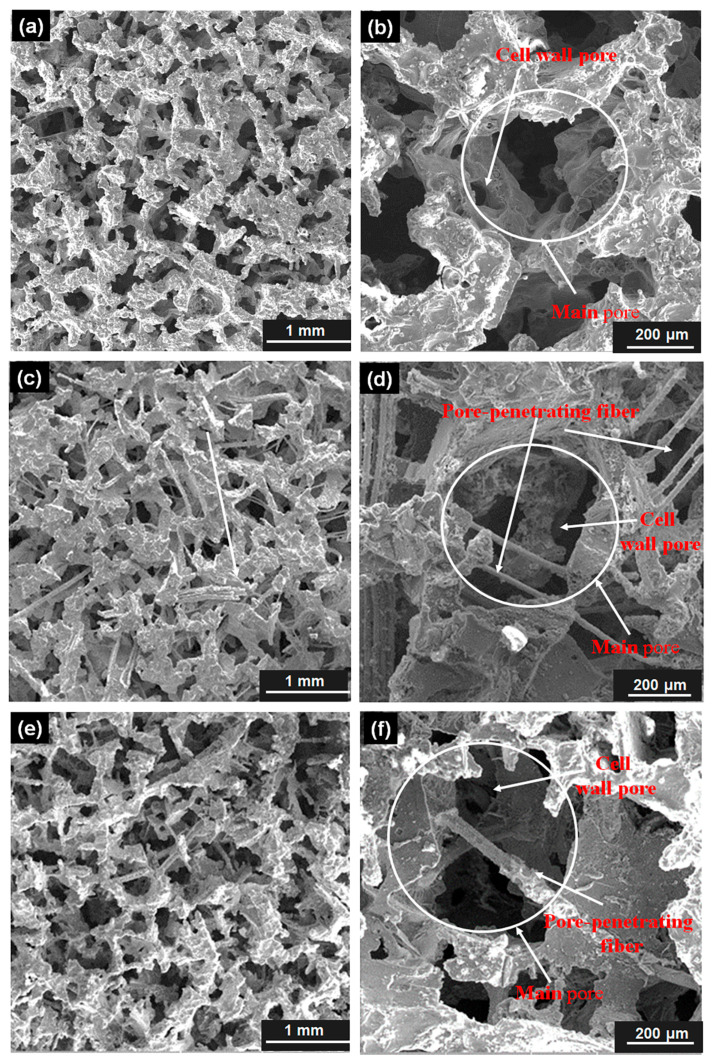
Pore structures of different aluminum foam samples with a pore size of 0.5 mm: (**a**,**b**) CIAF sample 6#; (**c**,**d**) PPFCAF sample 4# with a pore-penetrating fiber size of 15 μm; (**e**,**f**) PPFCAF sample 5# with a Pore-penetrating fiber size of 30 μm.

Figure 4 displays the pore structures of CIAF sample 6# and PPFCAF samples 4# and 5#. The pores in the aluminum foam samples formed interconnected polyhedra, while the PPFCAF samples showed a more regular pore pattern. This is because the pore cavities were derived from naturally crystallized polyhedral NaCl particles, making it challenging to produce a uniform surface morphology. Aluminum foam consisted of a single main pore and cell wall pores, with multiple cell wall pores within the voids. This structure formed when NaCl particles came into contact during cold pressing, creating unwet regions where molten aluminum could not fill the gaps during subsequent infiltration processes [23]. Compared to CIAF, PPFCAF features pore-penetrating fibers, with both ends of each fiber in contact with the pore walls, resulting in a fiber-pore interpenetrating network structure. When 2–3 smaller fibers were present, the pore-penetrating fibers of different sizes were aligned parallel to each other. This occurred because multiple pore-penetrating fibers came into contact during the preparation of the fiber–NaCl composite, and the smaller fiber size allowed NaCl crystallization to encapsulate several fibers fully. The pore structure parameters for each of the three samples were summarized in Table 2. The CIAF sample 6# and the PPFCAFs samples 4# and 5# had comparable average main pore sizes and average cell wall pore sizes, ranging from 581 μm to 537 μm and 223 μm to 252 μm, respectively.

#### 3.1.2. Effect of Pore Size on Structure

**Figure 5:**
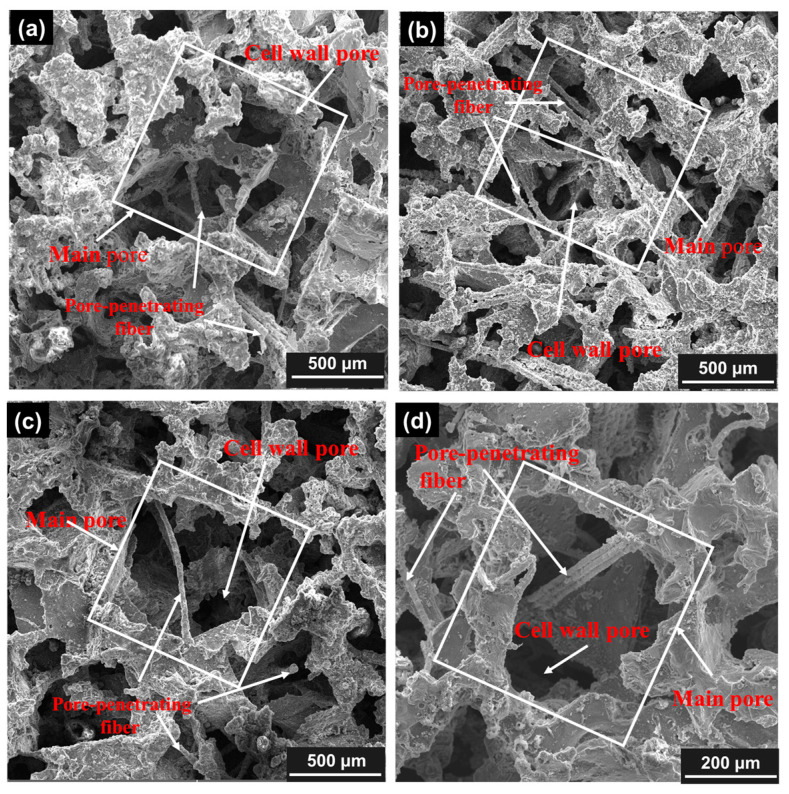
Pore structures of PPFCAFs with different pore sizes: (**a**) 0.8 mm pore size; (**b**) 0.7 mm pore size; (**c**) 0.6 mm pore size; (**d**) 0.5 mm pore size. (white boxes represent main pores).

Figure 5a–d depicts the pore structure morphology of samples 1#, 2#, 3#, and 4#, respectively. As the main pore size increased, the pore morphology, pore wall outlines, and fiber distribution became more distinct. Additionally, the main pore size affected the fiber content in PPFCAF. Smaller main pores in sample 4# contained more pore-penetrating fibers (refer to Table 1), leading to the highest fiber density within its pores. Since pore-penetrating fibers were linked to individual particles, a reduction in pore size resulted in more pores and more pore-penetrating fibers. This aspect partly impacted the sound absorption performance of PPFCAFS.

As presented in Table 3, the main pore sizes of samples 1#, 2#, 3#, and 4# of the PPFCAFs were 855 μm, 751 μm, 634 μm, and 537 μm, respectively. A decrease in pore size was accompanied by a reduction in cell wall pore size, with measured values of 398 μm, 340 μm, 262 μm, and 225 μm for samples 1# to 4#, respectively. Observation of Figure 5 shows that the number of pore walls ranges from 6 to 8, similar to that of CIAF.

### 3.2. Sound Absorption Performance

#### 3.2.1. Effect of Pore-Penetrating Fibers on Sound Absorption Curves

**Figure 6:**
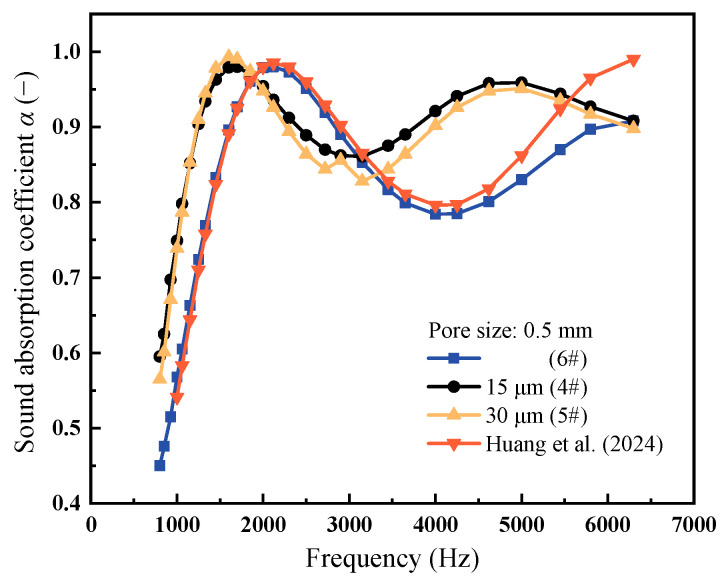
Sound absorption curves of CIAF, PPFCAFs, and SP-SS304FAF [16] with 0.5 mm pore size.

Figure 6 presents the sound absorption curves for CIAF and PPFCAF samples, with corresponding parameters provided in Table 4. Comparing the blue and black lines in Figure 6 shows that incorporating pore-penetrating fibers significantly enhanced the sound absorption performance. This enhancement was attributed to the increased flow resistivity and tortuosity listed in Table 5. The presence of pore-penetrating fibers raises both tortuosity and flow resistivity, thereby effectively enhancing sound absorption performance. Specifically, the PPFCAF sample 4# exhibited a sound absorption valley at 3150 Hz, absorbing 86% of incident sound energy—a 9.8% increase over CIAF. This result led to a 9.9% increase in the average sound absorption coefficient.

The performance gains were primarily due to viscous and thermal losses within porous materials. Pore-penetrating fibers increased the surface area and pore structural complexity of the pore. When sound waves enter these pores, the pore-penetrating fibers promote energy dissipation through viscous and thermal effects [24], thereby improving overall the material’s sound absorption performance. Compared with previous studies [16], the PPFCAFs produced by evaporation crystallization exhibited significantly higher average sound absorption coefficients and a sound absorption valley. This confirmed that the pore-penetrating fibers in the new structure significantly improved the aluminum composite foam’s ability to absorb sound.

Further analysis of the standing wave effect revealed that the first absorption peak corresponds to the fundamental frequency (first harmonic) at a quarter-wavelength resonance (approximately 1800 Hz), while the second absorption peak aligned with the third harmonic resonance (approximately 5400 Hz). The sound absorption valley occurred at the fundamental frequency (first harmonic) at a half-wavelength resonance (3600 Hz). It was observed that the sound absorption peaks and valleys of PPFCAFs shift toward lower frequencies. As indicated in Table 5, this shift indicated an increase in tortuosity, which reduced the effective sound speed within the porous structure, thereby lowering the quarter-wavelength resonance frequency.

Finally, comparing the sound absorption performance of PPFCAFs with different pore-penetrating fiber diameters showed that samples with 15 μm pore-penetrating fibers had higher sound absorption valley values. This suggests that more pore-penetrating fibers boost energy dissipation and loss. Therefore, this study demonstrates that the acoustic performance of foam structures can be improved by increasing the fiber density within the material pores.

**Figure 7:**
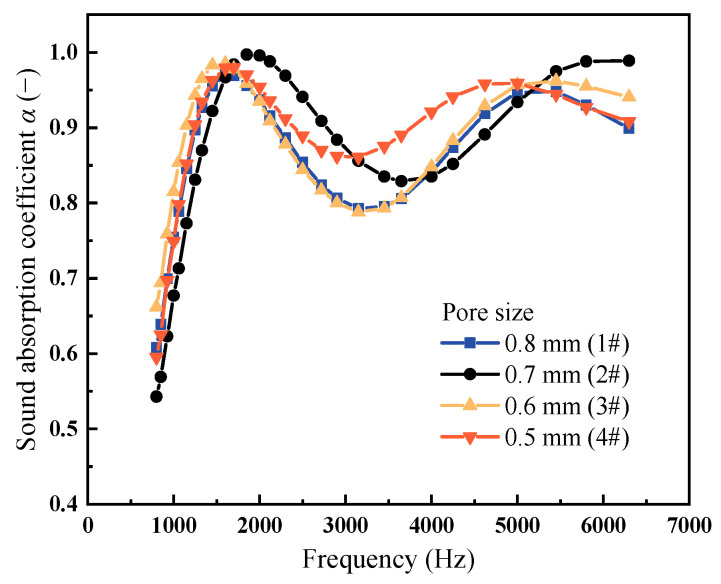
Sound absorption curves of PPFCAFs with different pore sizes.

Figure 7 compares the sound absorption curves of PPFCAFs with different pore sizes. The sound absorption valley value rose as the pore size fell. Compared to sample 1# with a 0.8 mm pore size, sample 4# with a 0.5 mm pore size exhibited a sound absorption valley value that increased from 0.713 to 0.861, representing a 20% improvement. In contrast, the sound absorption peak showed no significant change (Table 3). This modification resulted in a 4.7% increase in the average sound absorption coefficient of the PPFCAFs, from 0.841 to 0.881. As analyzed in Section 3.1, both the main pore size and pore-penetrating fiber content positively influenced the sound absorption of PPFCAF. At a constant porosity, decreasing the main pore size corresponds to a reduction in the wall thickness of pores. When sound waves pass through the foam, increased viscous effects and thermal conduction between the sound waves and pores convert more sound energy into heat, thereby enhancing sound absorption [25]. Additionally, pore-penetrating fibers (volume density) within the aluminum foam increased as the main pore size decreased. This elevated the material’s flow resistivity, enhanced viscous dissipation effects, and improved the sound absorption performance of the PPFCAFs [26].

Additionally, the highest flow resistivity is associated with the smallest average main pore size, further confirming that flow resistivity is a key factor affecting sound absorption performance.

#### 3.2.2. Acoustic Impedance

**Figure 8:**
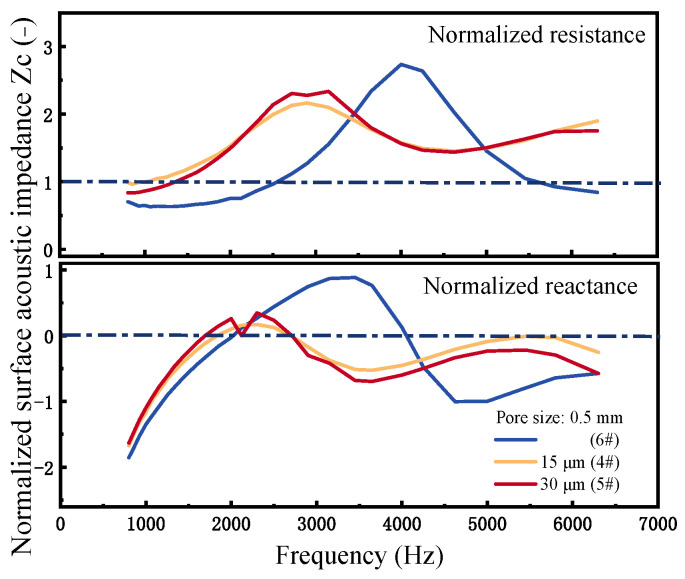
Surface acoustic impedance of CIAF with 0.5 mm pore size and PPFCAFs.

Acoustic impedance is a crucial factor in determining sound absorption and reflection in sound-absorbing materials. Figure 8 compares the acoustic impedance curves of PPFCAFs and CIAF. Its real part characterizes energy dissipation (associated with viscous and thermal losses), while the imaginary part (acoustic reactance) reflects energy storage capacity and the phase relationship between sound pressure and particle velocity, directly influencing the material’s reflection characteristics. Equation (16) establishes the relationship between acoustic impedance and sound absorption coefficient. Furthermore, this coefficient is determined by both the normalized surface acoustic resistance *Rs* and the normalized surface acoustic reactance *X_s_*, Optimal impedance matching with air is achieved when *R_s_* is close to 1, and *X_s_* approaches 0 [22].

(16) $$\alpha =\frac{4R_{s}}{{\left(1+R_{s}\right)}^{2}+X_{s}^{2}}$$

where *R_s_ = Re*(*Zc*)/(*ρ*_0_*c*_0_) represents the normalized surface acoustic resistance, *X_s_ = Im*(*Zc*)/(*ρ*_0_*c*_0_) denotes the normalized surface acoustic reactance, *Re*(*Zc*) and *Im*(*Zc*) represent the real and imaginary parts of the surface acoustic impedance of the sound-absorbing material. *ρ*_0_*c*_0_ is the acoustic impedance of air.

As shown in Figure 8, in the sound absorption peak region (800–2500 Hz), the normalized surface acoustic resistance *R_s_* was close to 1, and the acoustic reactance *X_s_* approached 0 (*R_s_* ≈ 1, *X_s_* ≈ 0) as the frequency increased, indicating that the material achieved impedance matching. In this condition, sound energy dissipation dominated while sound energy storage effects were negligible, thereby enabling highly efficient sound absorption. Among the samples, PPFCAFs 4# exhibited an acoustic resistance *R_s_* even closer to 1 and a reactance *X_s_* nearer to 0, suggesting better impedance matching. Consequently, sound waves effectively penetrated the material, and as the flow resistivity of the perforated fibers increased, sound energy dissipation further enhanced, improving the overall sound absorption performance.

In the sound absorption valley region (2500–5000 Hz), the magnitude change of the normalized surface acoustic reactance was greater than that of the resistance (∆X_s_ > ∆R_s_), indicating that the system primarily stored acoustic energy through elastic/inertial mechanisms, while viscous-thermal dissipation was relatively weak. This caused the acoustic impedance to deviate substantially from the matched condition (Zs = 1), leading to strong reflection and consequently minimizing the sound absorption coefficient.

In the sound absorption valley region (2500~5000 Hz), the normalized surface acoustic resistance *R_s_* of the CIAF sample 6# remained consistently above 1.5 with increasing frequency, while its acoustic reactance *X_s_* oscillated between −1 and 1. This indicated that this region was simultaneously affected by high impedance mismatch and phase disturbances induced by the multi-scale structure. Although the material remained in a dissipation-dominant state, the ineffective penetration of sound waves resulted in low absorption coefficients.

In contrast, the PPFCAF sample 4# exhibited a normalized surface acoustic resistance close to 1 across this region. The magnitude of its normalized acoustic reactance was small and changed gradually (Xs = −0.8~0.5). This demonstrated that the composite structure provided improved impedance matching, effectively reducing sound wave reflection and suppressing localized standing waves caused by phase disturbances. Consequently, a greater proportion of sound waves could enter the material’s interior. Furthermore, the high flow resistivity and tortuosity introduced by the perforated fibers enhance the viscous-thermal dissipation capability. The synergistic effect of these two mechanisms significantly improved the sound absorption performance within the sound valley region.

Additionally, research indicated that PPFCAFs exhibited little difference in acoustic impedance across varying fiber diameters. When analyzing this phenomenon, both diameters and fiber content must be taken into account. Given the minimal variation in these two variables, their influence on sound absorption performance was limited.

**Figure 9:**
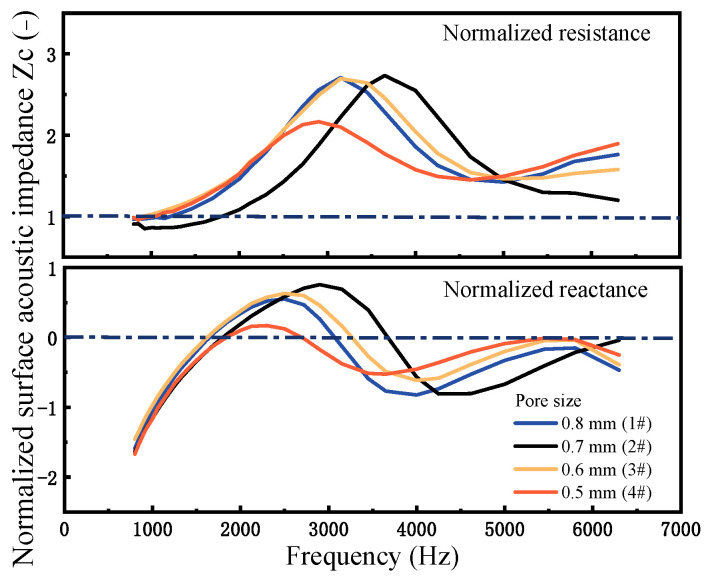
Surface acoustic impedance of PPFCAFs with different pore sizes.

Figure 9 compares the normalized surface acoustic impedance of perforated-fiber-reinforced aluminum foams with different main pore diameters. In the absorption peak region (800–2500 Hz), decreasing the main pore diameter leads to an increase in the normalized resistance (R > 1) and a greater deviation of the normalized reactance from zero, indicating reduced impedance matching—manifested as a slight decrease in the absorption peak. In contrast, within the sound absorption valley range (2500–5000 Hz), reducing the main pore diameter brought the normalized resistance closer to 1 and the normalized reactance closer to 0, signifying improved impedance matching with that of air. Under this condition, sound reflection was significantly suppressed, standing waves induced by phase perturbations were weakened, and more sound energy penetrated into the material. Concurrently, the moderately increased flow resistivity enhanced viscous dissipation, thereby achieving higher sound absorption performance in the valley frequency band. These findings are consistent with the absorption coefficient results shown in Figure 7.

## 4. Finite Element Analysis

Based on the research in Section 3.1 and Section 3.2, pore-penetrating fibers have a positive impact on the sound absorption performance of PPFCAF. At present, analyzing sound absorption performance from the perspective of sound absorption is a conventional research approach. However, it is insufficient for the sound absorption valley that we are concerned about. As the frequency corresponding to the sound absorption valley (where the 1/2 sound wavelength is an even multiple), the reflected sound wave is significantly enhanced, directly interfering with the effective absorption of the incident sound wave. Therefore, it is necessary to comprehensively analyze its acoustic characteristics from sound absorption and reflection perspectives.

### 4.1. Equivalent Fluid Model

**Figure 10:**
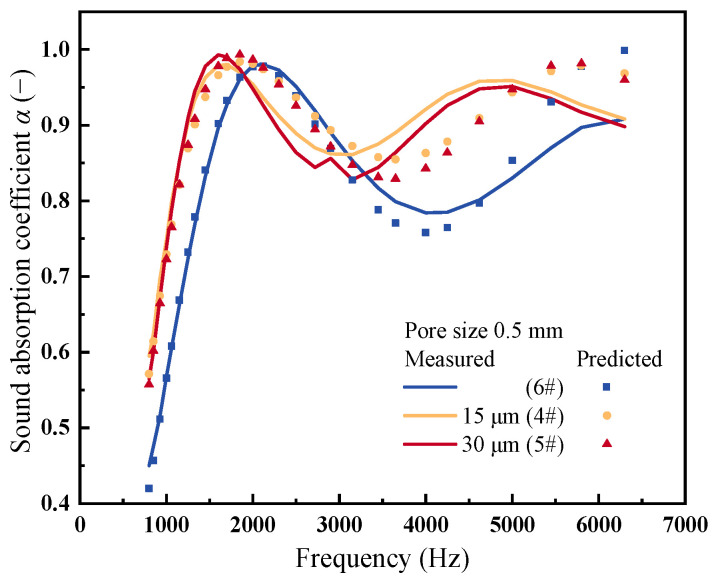
Predicted sound absorption curves for CIAF and PPFCAF.

**Figure 11:**
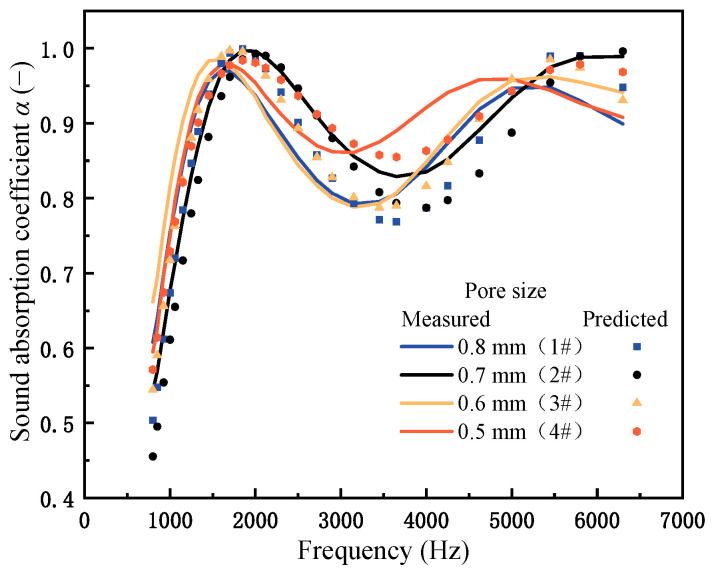
Predicted sound absorption curves for PPFCAFs with different pore sizes.

The equivalent fluid model utilized the Wilson model as the theoretical acoustic model and simulated acoustic characteristics, including the sound absorption curve, reflected sound pressure field, and power consumption of PPFCAFs. Figure 10 compares the measured and simulated sound absorption curves of CIAF with a main pore size of 0.5 mm and PPFCAFs. Figure 11 compares the measured and simulated sound absorption curves of PPFCAFs with different main pore sizes. It could be seen that there was a good agreement between the predicted values and the simulated values of the sound absorption curve. In subsequent research, the Wilson acoustic model was used as the physical model, combined with finite element simulation, to analyze the reflected sound pressure and sound power density of the PPFCAFs, thereby further studying their sound absorption behavior at the sound absorption valley frequency.

**Figure 12:**
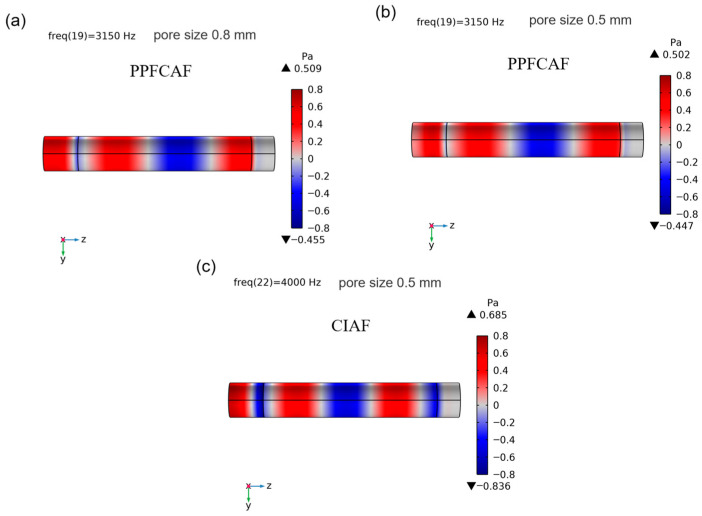
Sound pressure level scatter for PPFCAFs and CIAF (**a**) PPFCAF with 0.8 mm pore size; (**b**) PPFCAF with 0.5 mm pore size; (**c**) CIAF with 0.5 mm pore size.

The frequency range of the sound absorption valley is a vital factor influencing the overall sound absorption performance, as detailed in Section 3.2 of the sound absorption performance study. Figure 12 illustrates the reflected sound pressure field within the absorption valley region for PPFCAF samples (designated as samples 1# and 4#), which possess similar porosities but differ in pore sizes of 0.8 mm and 0.5 mm, respectively, as well as for a CIAF sample 6# with a pore size of 0.5 mm. As observed in Figure 12, the reflected sound pressure of the CIAF sample #6 exhibited phase inversion (changing from red to blue) and formed a strong sound pressure amplitude (weak sound velocity amplitude) on the surface. This indicated that the reflected wave formed a standing wave on the material surface, with a reflected sound pressure range of −0.455 to 0.509 Pa. This suppresses surface particle vibration, preventing sound waves from effectively penetrating the material. Sample 4# of the perforated fiber-reinforced foam aluminum composite exhibited no phase inversion (remaining red throughout with only a slight blue transition at the edges) and showed no significant sound pressure amplitude. Its narrow sound pressure amplitude range (−0.447 Pa to 0.502 Pa) indicated minimal particle vibration velocity at the surface, allowing partial dissipation of sound waves through air particles.

At the sound absorption valley frequency, the overall amplitude range of the reflected sound pressure field decreased due to the presence of pore-penetrating fibers. As the pore size decreased, the amplitude range of the reflected sound pressure field also reduced accordingly. The reflected sound pressure amplitude range of the PPFCAF sample 4# with a main pore size of 0.5 mm was the minimum. This further substantiates the evidence that composite foam aluminum incorporating perforated fibers demonstrates diminished reflectivity within the sound absorption valley.

At the frequency corresponding to the sound-absorbing valley, the incident wave and reflected wave undergo destructive interference, with the sound reflection effect becoming dominant. This means that incident sound waves are heavily reflected, and only a tiny percentage is absorbed by the porous material. Therefore, the sound absorption valley value is affected by the degree of sound reflection. Furthermore, the structure’s tortuosity increases due to pore-penetrating fibers inside the pores. As sound travels through the porous structure, the pore-penetrating fibers reduce the reflection of sound, progressively decreasing the range of reflected sound pressure amplitudes and increasing the sound absorption valley value.

Furthermore, Figure 12a,b reveal that as the main pore size decreased, the phase shift of reflected acoustic waves diminished, and the overall amplitude range of the sound pressure field narrowed, with a more confined sound pressure amplitude distribution. The standing wave effect weakened. In the sound absorption valley, as the main pore size decreased, the complex pore structure enhanced the flow resistivity and tortuosity (Table 5), resulting in a decrease in the material’s surface acoustic reactance (Figure 9), thereby attenuating sound wave reflection.

**Figure 13:**
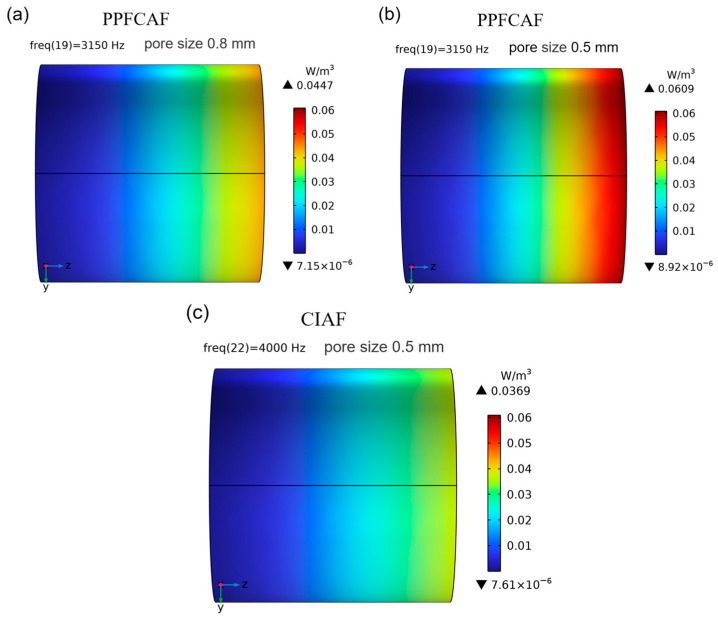
Total acoustic power dissipation of PPFCAFs and CIAF: (**a**) PPFCAF with 0.8 mm pore size; (**b**) PPFCAF with 0.5 mm pore size; (**c**) CIAF with 0.5 mm pore size.

Figure 13 compares the total sound power dissipation density of PPFCAFs and CIAF with different pore sizes at the absorption valley frequency. Notably, reflection is the primary mechanism in the sound-absorbing valley region. Reducing the sound reflection coefficient will weaken the standing wave effect, which is a key condition for achieving efficient sound absorption in the valley. As observed in Figure 13, increasing fiber content enhanced sound wave disturbance within the air medium, further improving sound absorption. A decrease in pore size led to an increase in sound power dissipation density. Sample 4#, showed the highest total sound power dissipation density at 0.0609 W/m^3^. This result indicates that reducing main pore size enhanced sound absorption across the sound absorption valley frequency range. Combined with analyses of acoustic impedance and reflected sound pressure, smaller main pore sizes result in reduced sound reflection and increased sound absorption. Furthermore, increased pore-penetrating fiber content partially enhances the disturbance of sound waves within the air medium, further strengthening sound absorption. Sample 4#, with a 0.5 mm pore size, exhibited the highest total sound power dissipation density at 0.0609 W/m^3^. This analysis also confirms the enhanced sound absorption effect of perforated fibers and small main pore size in the absorption valley region.

### 4.2. Ideal Geometric Model

**Figure 14:**
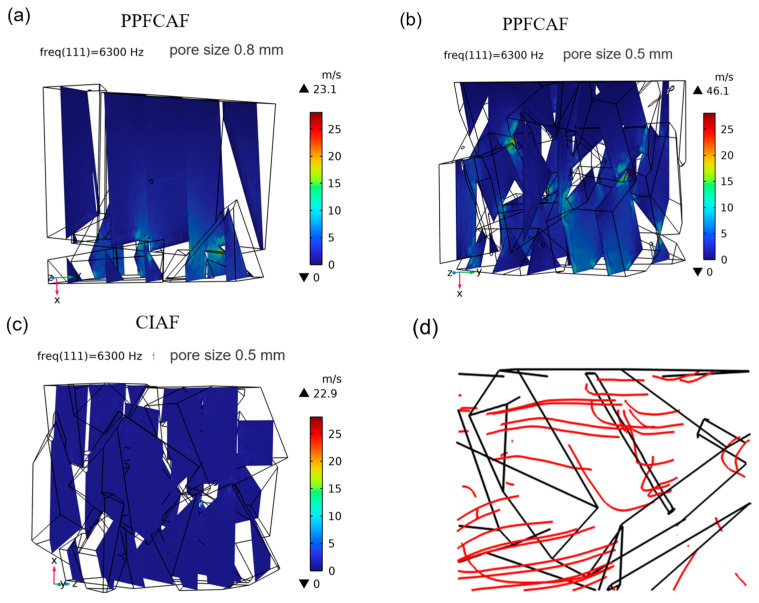
Variations in sound particle velocity for PPFCAFs and CIAF: (**a**) PPFCAF with 0.8 mm pore size; (**b**) PPFCAF with 0.5 mm pore size; (**c**) CIAF with 0.5 mm pore size; (**d**) Sound rays undergoing flow deflection due to fiber influence.

Figure 14 displays the instantaneous total sound particle velocity distribution across three-dimensional geometries for PPFCAFs with main pore sizes of 0.8 mm and 0.5 mm, and CIAF with a main pore size of 0.5 mm. The PPFCAFs had a higher instantaneous total sound velocity than CIAF, with the PPFCAF reaching 46.1 m/s. Figure 14d presents a post-processed visualization of the acoustic particle velocity field obtained from finite element simulations, depicting the oscillatory motion of air particles driven by acoustic excitation. It illustrates the oscillatory motion of air particles as sound waves propagate through the porous structure and interact with penetrating fibers. As seen in Figure 14d, pore-penetrating fibers complicate the path of sound waves within the material, causing sound streamlines to flow around the pore-penetrating fibers. This effectively increases the propagation distance of sound waves, leading to greater absorption during transmission.

Additionally, the instantaneous total sound particle velocity within the PPFCAF increased as the main pore size decreased. With smaller main pores, the pore structure becomes more intricate, especially as narrow channels speed up fluid flow. This alteration enhances the interaction between sound waves and the air medium during propagation within the pores, resulting in more vigorous acoustic energy dissipation within the material.

**Figure 15:**
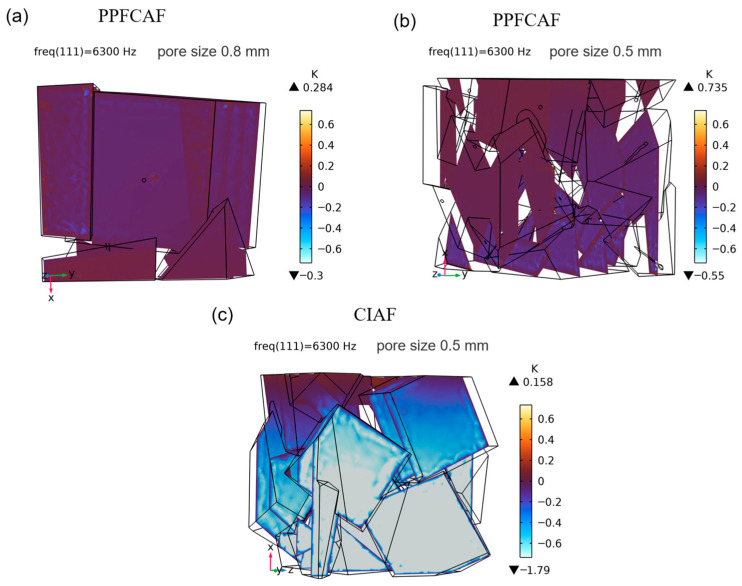
Temperature field of PPFCAFs and CIAF: (**a**) PPFCAF with 0.8 mm pore size; (**b**) PPFCAF with 0.5 mm pore size; (**c**) CIAF with 0.5 mm pore size.

Figure 15 illustrates the temperature distribution within the three-dimensional geometry of the PPFCAFs with main pore sizes of 0.8 mm and 0.5 mm, as well as the CIAF with a main pore size of 0.5 mm, all of which are maintained at the same porosity. As evidenced in Figure 15, the PPFCAF (sample 4#) demonstrated a thermal field that was 0.735 K higher than that of the CIAF under equivalent acoustic excitation, indicating an improved thermoacoustic coupling efficiency. Thermal phenomena drive this performance: pore-penetrating fibers enhance velocity gradients, thereby increasing viscous dissipation through boundary layer acceleration. As the main pore size diminishes, the heat exchange among the acoustic wave, air, and the pore is improved, given that the main pore size governs the heat exchange efficiency. Furthermore, the heat dissipation of the acoustic wave was subsequently increased.

## 5. Sound Absorption Enhancement Mechanism of PPFCAF

In conclusion, experiments and finite element simulations show that the sound absorption capabilities of PPFCAF are influenced by both the pore-penetrating fibers and the main pores size. The mechanism analysis is as follows.

### 5.1. The Influence of Pore-Penetrating Fibers

The introduction of pore-penetrating fibers increases the propagation path of sound waves within the pore structure, thereby enhancing tortuosity (Table 5) and lowering the frequency of the first resonance peak. Meanwhile, the increased specific surface area of the pore raises the flow resistivity (Table 5), leading to an overall improvement in sound absorption performance. Notably, in the sound absorption valley frequency range (2500–5000 Hz)—compared to the sound absorption peaks—the incorporation of pore-penetrating fibers effectively elevates the absorption valley. Impedance measurements (Figure 8) reveal that the addition of perforated fibers reduces the normalized surface resistance, thereby weakening acoustic reflection caused by impedance mismatch. This is further corroborated by finite element simulations, which show a reduced reflected sound pressure amplitude ranging from–0.470 Pa to 0.502 Pa (Figure 12b). As a result, more sound energy effectively enters and propagates within the material.

The introduction of perforated fibers effectively increases the specific surface area of the pore channels [27], thereby enhancing the static flow resistivity of the pore structure. Additionally, the perforated fibers elongate the sound propagation path (Figure 4d), increasing tortuosity (Table 5). Together, these effects strengthen both viscous and thermal dissipation mechanisms, resulting in a total acoustic power dissipation density of 0.0609 W/m^3^ in the material (Figure 13).

### 5.2. The Influence of the Main Pore Size

As the main pore diameter decreases, the static flow resistivity and tortuosity of the pore structure increase (Table 5), leading to an overall improvement in sound absorption performance, particularly in the absorption valley values (rising from 0.713 to 0.861). According to the acoustic impedance measurements (Figure 9), within the absorption valley range (2500–5000 Hz), the normalized surface resistance approaches 1, and the normalized surface reactance approaches 0, indicating a gradual matching of the acoustic impedance with that of air. Under these conditions, the effect of sound energy storage is weakened, significantly reducing sound wave reflection (Figure 11) and allowing more sound energy to penetrate the material. Simultaneously, the moderately increased flow resistivity and tortuosity enhance the material’s viscous dissipation (from 28.1 m/s to 46.1 m/s) and thermal losses (from 0.284 K to 0.735 K). Finite element simulations reveal that the total acoustic power dissipation density increases from 0.0447 to 0.0609 W/m^3^. Consequently, this results in higher sound absorption performance within the absorption valley frequency band.

## 6. Conclusions

This study proposes a precursor fabrication approach for creating composite aluminum foam with a pore-penetrating fiber network through infiltration casting. The PPFCAF was investigated through experimental and finite element numerical simulation. The combination effect of the pore-penetrating microfibers and refined pore cavities improves the sound absorption performance. Results indicate that the PPFCAF achieves a sound absorption valley value of 0.861 and an average sound absorption coefficient of 0.880 across the frequency range of 800–6300 Hz. This performance outperforms CIAF (by 9.8% and 9.9%, respectively). Pore-penetrating fibers and small main pores enhance the flow resistivity and tortuosity of the pore structure and increase the viscous–thermal dissipation of sound waves within the material. Acoustic impedance tests indicate that the PPFCAF is primarily dominated by acoustic energy dissipation. Within the absorption valley frequency range (2500–5000 Hz), the normalized surface impedance more closely approaches the characteristic impedance of air, indicating improved local impedance matching. This reduces standing wave effects caused by sound reflection and facilitates greater penetration of sound energy into the material. Meanwhile, increased flow resistivity and tortuosity promote efficient dissipation of sound energy within the material, collectively enhancing the overall sound absorption performance across the entire frequency band. This novel perforated fiber composite structure not only holds significant potential for development and application in the field of sound absorption but also shows promise in areas such as wave interference.

## Figures and Tables

**Table 1:** Samples pore structure parameters of PPFCAFs.

Sample	Porosity *ϕ*/%	Fiber Diameter *d_f_*/mm × Length *L_f_*/mm	Main Pore Size of the Sample *D*/mm
1#	82.4	Φ0.015 × 3	0.8
2#	82.3	Φ0.015 × 3	0.7
3#	81.8	Φ0.015 × 3	0.6
4#	82.8	Φ0.015 × 3	0.5
5#	82.3	Φ0.030 × 3	0.5
6#	82.1	-	0.5

**Table 2:** Content of fibers in fiber–NaCl composite particles.

NaCl Particle Size *D_NaCl_*/mm	Fiber–NaCl Composites Mass $M_{t}$/g	Fiber Mass $M_{f}$/g	Fiber Content *V_f_*/vol	Fibers Numbers *N/−*
0.8	5	0.1207	0.85%	109,483
0.7	5	0.1197	0.84%	124,156
0.6	5	0.1108	0.78%	134,042
0.5	5	0.0951	0.67%	138,058

**Table 3:** Pore structure parameters of PPFCAFs.

Samples	Fiber Diameter *r*/μm	Porosity *ϕ*/%	Average Main Pore Size *D*/μm (Standard Deviation σ)	Average Cell Wall Pore Size *d*/μm (Standard Deviation σ)
1#	15	82.4	855 (0.073)	398 (0.045)
2#	15	82.3	751 (0.058)	340 (0.082)
3#	15	81.8	634 (0.060)	262 (0.059)
4#	15	82.8	537 (0.043)	225 (0.044)
5#	30	82.3	581 (0.051)	252 (0.053)
6#	-	82.1	545 (0.086)	223 (0.038)

**Table 4:** Sound absorption performance parameters of CIAF and PPFCAFs.

Sample	Porosity /%	Sound Absorption Peak Frequency/Hz	Sound Absorption Peak Value/−	Sound Absorption Valley Frequency /Hz	Sound Absorption Valley Value/−	Average Sound Absorption Coefficient α (800~6300 Hz)/−	Average Sound Absorption Coefficient α (1000~6300 H)/−
1#	82.4	1450	0.974	3150	0.713	0.841	0.882
2#	82.3	1850	0.999	3650	0.785	0.862	0.892
3#	81.8	1600	0.985	3150	0.788	0.876	0.897
4#	82.8	1700	0.980	3150	0.861	0.881	0.909
5#	82.3	1600	0.993	3150	0.828	0.870	0.901
6#	82.1	2120	0.980	4000	0.784	0.801	0.839
Ref [16]	82.0	2000	0.985	4250	0.797	—	0.849

**Table 5:** Acoustic parameters of PPFCAFs and CIAF.

Sample	Fiber Diameter *r*/μm	Porosity *Φ*/%	Tortuosity *α_∞_*/−	Flow Resistivity *σ_f_*/Pa·s/m^2^
1#	15	82.4	1.763	17,863
2#	15	82.3	1.521	18,148
3#	15	81.8	1.811	21,723
4#	15	82.8	1.986	29,215
5#	30	82.3	1.535	25,335
6#	**−**	82.1	1.506	12,426

## Data Availability

The original contributions presented in this study are included in the article. Further inquiries can be directed to the corresponding authors.

## Supplementary Materials

The following supporting information can be downloaded at: https://www.mdpi.com/article/10.3390/ma18245515/s1, Figure S1: Flow resistivity testing device; Figure S2: Acoustic impedance testing system.

## Nomenclature

The following symbols are used in this manuscript:

***Symbol***	**Meaning**	**Unit/Notes**
*c_0_*	Sound speed	m/s
*c_p_*	Specific heat at constant pressure	J/(kg·K)
*d*	Average cell wall pore diameter	mm
*D*	Average main pore diameter	mm
*I*	Dentity tensor	Dimensionless
*Im(ρ_eq_)*	Imaginary part of equivalent density	kg/m^3^
*j*	Imaginary unit	Dimensionless
*Hi*	Incident wave transfer function	Dimensionless
*Hr*	Reflected wave transfer function	Dimensionless
*H*_12_	Sound pressure transfer function	Dimensionless
*K_eq_*	Equivalent bulk modulus	Pa
*K_C_*	Complex wave number	rad/m
*K_∞_*	Bulk modulus of high frequency limit	Dimensionless
*k*	Thermal conductivity	W/(m·K)
*L*	Thickness of the sample	mm
*L_f_*	Fiber length	mm
*Mf*	Mass of fiber	g/kg
*Mt*	Mass of fiber–NaCl particle composite	g/kg
*N*	Fibers numbers	μm
*p*_0_	Air of basic state pressure	Pa
*p_t_*	Pressure perturbation (The part varying with time). The total temperature is p = *p*_0_ + *p_t_*	Pa
*Pr*	Prandtl constant	Dimensionless
*Q*	Volumetric heat source term	W/m^3^
*q_v_*	Air flow rate	m/s
*r*	Fiber diameter	μm
*R*	Sound reflected coefficient	Dimensionless
*Re(ρ_eq_)*	Real part of equivalent density	kg/m^3^
*R_s_*	Normalized surface acoustic resistance	Dimensionless
*x_1_*	microphone and measured the distance between the samples	m
*X_s_*	Normalized surface acoustic reactance	Dimensionless
*T*	Transpose symbol	Matrix operation
*T*_0_	Basic state temperature/air temperature at standard temperature	K
*T_t_*	Temperature density perturbation (the part varying with time). The total temperature is *T* = *T*_0_ + *T_t_*	K
**u_t_**	air velocity vector	m/s
*Zc*	Surface acoustic impedance	kg/(m^2^·s)
*α*	Sound absorption coefficient	Dimensionless
*α_p_*	Thermal expansion coefficient	K^−1^
*α_∞_*	Tortuosity	Dimensionless
*β_T_*	Isothermal compressibility, βT = 1/p0	Pa^−1^
*γ*	Specific heat ratio	Dimensionless
*μ*	Dynamic viscosity of air	Pa·s
*μ_B_*	Bulk viscosity of air	Pa·s
*ρ*_0_	Air density at standard temperature and pressure	kg/m^3^
*ρ_t_*	Air density perturbation (the part varying with time). The total density is *ρ* = *ρ*_0_ + *ρ_t_*	kg/m^3^
*ρ_∞_*	High-frequency dynamic density	kg/m^3^
*ρ_eq_*	Equivalent density	kg/m^3^
*ρ_f_*	Density of 316L stainless steel fiber	7.80 g/cm^3^
*ρ_NaCl_*	Density of NaCl particles	2.167 g/cm^3^
*ρ_AlSi12_*	Density of AlSi12 aluminum foam	2.55 g/cm^3^
*σ_f_*	Flow resistance	Dimensionless
*ϕ*	Foam porosity	Dimensionless
*τ_vor_*	Vortex mode relaxation time	s
*τ_ent_*	Entropy mode relaxation time	s
*Δp*	Pressure drop	Pa
*ω*	Angular frequency	rad/s
∇	Gradient operator (Nabla operator)	Nabla operator
