# Supplementary material for: Enhanced Sound Absorption of Aluminum Foam Composites by Introducing Pore-Penetrating Fibers
Source: Materials (Basel). 2025 Dec 8;18(24):5515. doi: 10.3390/ma18245515 (PMC12734820; doi:10.3390/ma18245515)
Supplement: Supplementary file 1 [file materials-18-05515-s001.zip › materials-3982889-supplementary.pdf]

Supplementary Materials

1. Flow Resistivity Testing

Flow resistivity is one of the key factors influencing the sound absorption performance of porous materials. It characterizes the viscous inertia effect of porous foam structures to a certain extent. Figure S1 shows a self-made static flow resistance testing apparatus. The experimental setup consists of a pressure reducing valve, flow control valve, flow meter, differential pressure gauge, and test tube. This test was conducted under the Chinese national standard GB/T 25077-2010.

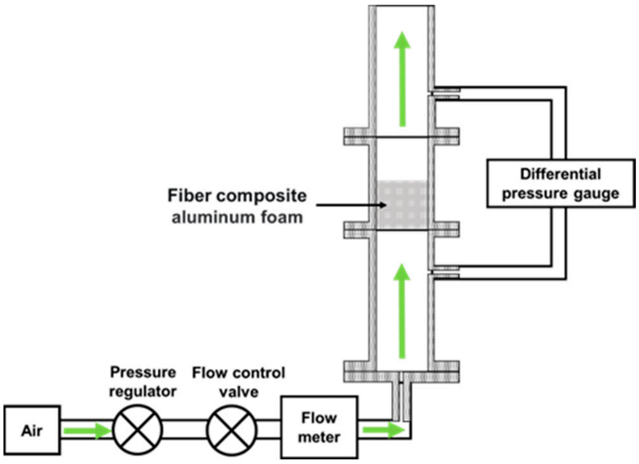

Figure S1. Flow resistivity testing device.

A compressed air source was used as the fluid medium, with air injection precisely controlled by a gas pressure reducer and flow control valve. The sample was placed within the test apparatus to ensure the differential pressure gauge monitors changes in air flow across both sides of the specimen. To characterize the flow resistivity under laminar flow conditions, the compressed air flow rate was controlled between 1.15 L/min and 4.15 L/min. The air flow rate was incremented by 0.3 L/min, with each increment repeated twice to record the flow rate q_v and the corresponding pressure drop Δp. The flow resistivity σ_f of the specimen was calculated from the pressure drop and flow rate, as shown in Equation (S1).

$$\sigma_f = \frac{\Delta p \pi L}{4q_{[illegible]}} \quad \text{(S1)}$$

Where, L represents the thickness of the sample.

2. Calculation of tortuosity

This study employed the extended functionality of the acoustic impedance tube testing system to measure the complex characteristic impedance Z_C and complex wave number K_C of the sample. Subsequently, the real part $Re(\rho_{eq})$ and imaginary part $Im(\rho_{eq})$ of the sample's equivalent density ρ_{eq} were calculated using Equation (S2). The theoretical tortuosity α_∞ was then computed by Johnson's equivalent density inversion Equation (S3).

$$\rho_{eq} = \frac{Z_C K_C}{\omega} \quad \text{(S2)}$$

$$\alpha_\infty = \frac{\phi}{\rho_0}\left(\mathrm{Re}\left(\rho_{eq}\right) - \sqrt{\left(Im\left(\rho_{eq}\right)\right)^2 - \left(\frac{\sigma_f}{\omega}\right)^2}\right) \quad \text{(S3)}$$

3. Sound absorption performance test

Impedance tube method is the most widely used method for measuring sound absorption properties of porous materials. In accordance with GB/T 18696.2-2002, the transfer function method was employed using an acoustic impedance tube (SW477, BSWATECH, Beijing, China) to evaluate the PPFCAFs, as illustrated in Figure S3.

The measurement system comprises an impedance tube (30mm diameter), two microphones, an audio amplifier, a multi-channel noise analyzer and analysis software. The test frequency range was 800-6300 Hz. The cylindrical sample is placed in the impedance tube, and the sample had no gap contact with the tube wall, and was a rigid backing. By measuring the sound pressure of two fixed microphones and calculating the transfer function with the multi-channel noise analyzer, the sound reflection coefficient *R* was obtained, and the Equation (S4) is as follows:

$$R = \frac{H_{12} - H_i}{H_r - H_{12}} e^{2jK_c x_1} \quad \text{(S4)}$$

Where H_i and H_r as the incident wave and reflected wave transfer function, H_{12} for sound pressure transfer function, for the wave number K_C, x_1 microphone and measured the distance between the samples, according to the Equation (S5) can be calculated the sound absorption coefficient of the sample.

$$\alpha = 1 - \left|R^2\right| \quad \text{(S5)}$$

The sound absorption coefficient, acoustic impedance of each sample were recorded following two measurement periods. Each sample underwent 3 measurements, and the average value was calculated. From the resulting sound absorption curve, the maximum value in the range within the 800~2500 Hz is determined was as the sound absorption peak value, while the minimum value in the range of 2500~5000 Hz was identified as the sound absorption valley value. In addition, the average sound absorption coefficient was determined by calculating the average sound absorption curve over the 800~6300 Hz range.

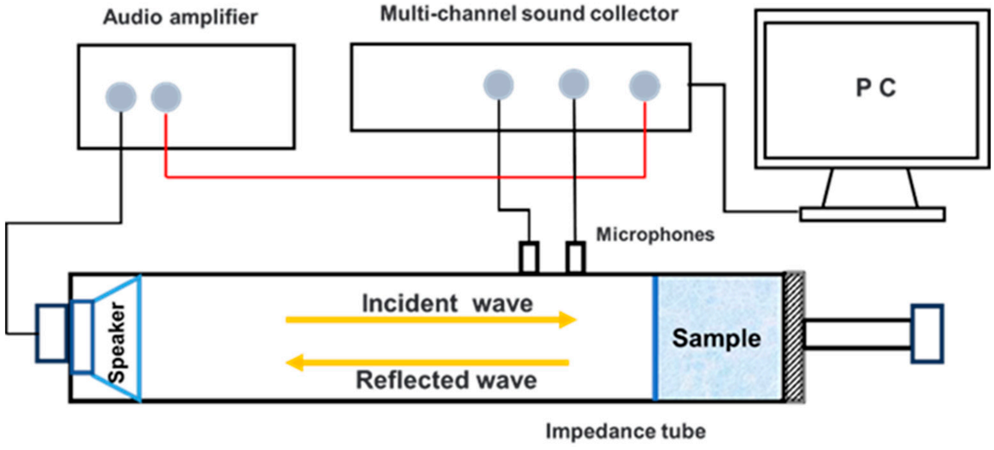

Figure S2. Acoustic impedance testing system
